# Engineering the *Oryza sativa* cell wall with rice NAC transcription factors regulating secondary wall formation

**DOI:** 10.3389/fpls.2013.00383

**Published:** 2013-10-01

**Authors:** Kouki Yoshida, Shingo Sakamoto, Tetsushi Kawai, Yoshinori Kobayashi, Kazuhito Sato, Yasunori Ichinose, Katsuro Yaoi, Miho Akiyoshi-Endo, Hiroko Sato, Tadashi Takamizo, Masaru Ohme-Takagi, Nobutaka Mitsuda

**Affiliations:** ^1^Technology Center, Taisei CorporationYokohama, Japan; ^2^Bioproduction Research Institute, National Institute of Advanced Industrial Science and TechnologyTsukuba, Japan; ^3^Japan Bioindustry AssociationTsukuba, Japan; ^4^Green Sogna Inc.Tsukuba, Japan; ^5^National Institute of Crop ScienceTsukuba, Japan; ^6^National Institute of Livestock and Grassland Science, National Agriculture and Food Research OrganizationNasushiobara, Japan; ^7^Institute for Environmental Science and Technology, Saitama UniversitySaitama, Japan

**Keywords:** NAC trascription factor, secondary cell wall, rice, genetic modification, CRES-T

## Abstract

Plant tissues that require structural rigidity synthesize a thick, strong secondary cell wall of lignin, cellulose and hemicelluloses in a complicated bridged structure. Master regulators of secondary wall synthesis were identified in dicots, and orthologs of these regulators have been identified in monocots, but regulation of secondary cell wall formation in monocots has not been extensively studied. Here we demonstrate that the rice transcription factors SECONDARY WALL NAC DOMAIN PROTEINs (SWNs) can regulate secondary wall formation in rice (*Oryza sativa*) and are potentially useful for engineering the monocot cell wall. The *OsSWN1* promoter is highly active in sclerenchymatous cells of the leaf blade and less active in xylem cells. By contrast, the *OsSWN2* promoter is highly active in xylem cells and less active in sclerenchymatous cells. *OsSWN2* splicing variants encode two proteins; the shorter protein (OsSWN2S) has very low transcriptional activation ability, but the longer protein (OsSWN2L) and OsSWN1 have strong transcriptional activation ability. In rice, expression of an OsSWN2S chimeric repressor, driven by the *OsSWN2* promoter, resulted in stunted growth and para-wilting (leaf rolling and browning under normal water conditions) due to impaired vascular vessels. The same OsSWN2S chimeric repressor, driven by the *OsSWN1* promoter, caused a reduction of cell wall thickening in sclerenchymatous cells, a drooping leaf phenotype, reduced lignin and xylose contents and increased digestibility as forage. These data suggest that OsSWNs regulate secondary wall formation in rice and manipulation of OsSWNs may enable improvements in monocotyledonous crops for forage or biofuel applications.

## INTRODUCTION

The secondary wall is a plant-specific, stiff and thick cell wall composed of lignin, cellulose and hemicelluloses in a complicated bridged structure. The secondary wall is synthesized just outside the plasma membrane of vascular vessels and fiber cells in xylem, which require mechanical rigidity and stability to retain water in the vessel, endure the internal pressure of water transport and support the plant’s aerial structures. Some species also produce secondary walls in the internal cell layer of anthers and siliques; the different shrinkage rates of cell layers with or without secondary walls produces tension that promotes dehiscence of anthers and siliques during dehydration (Keijzer, 1987; Mitsuda and Ohme-Takagi, 2008). In contrast to dicots and conifers, monocotyledonous plants do not possess vascular cambium but do develop secondary walls within the leaf blade and culm, and in vascular vessels. However, the composition of monocot secondary cell walls differs from that of dicots (Carpita and Gibeaut, 1993; Yokoyama and Nishitani, 2004) and the developmental regulation of secondary wall formation in monocots has not been well characterized, except for identification of some enzymes and related proteins (Li et al., 2003; Hirano et al., 2010; Kotake et al., 2011).

Some secondary cell wall components can negatively affect downstream uses of plant biomass. For example, monocots are traditionally important forage crops; however, high lignin content results in poor digestibility. Also, several monocot plants, such as miscanthus and switchgrass, have recently emerged as potential feedstock crops for cellulosic biofuels (Schmer et al., 2008); however, high lignin can adversely affect the conversion of cellulose to fermentable sugars. In addition, high arabinoxylan and xylan contents in primary and secondary cell walls of monocots may require an additional xylose fermentation after saccharification. By contrast, pyrolytic biofuels applications may benefit from high lignin content, which increases energy density. Therefore, elucidation of the mechanisms regulating secondary wall biosynthesis in monocots has the potential to improve crops for multiple important applications.

The regulation of secondary wall formation has been extensively studied in the model dicot *Arabidopsis thaliana*. These studies revealed that a specific set of NAC transcription factors acts as master regulators of vascular vessel differentiation and secondary wall formation in fiber cells (Kubo et al., 2005; Mitsuda et al., 2005, 2007; Zhong et al., 2006; Ko et al., 2007; Mitsuda and Ohme-Takagi, 2008). For example, the expression of chimeric repressors of some *VASCULAR ASSOCIATED NAC DOMAIN PROTEIN* (*VND*) genes inhibited the differentiation of vascular vessels (Kubo et al., 2005). Also, the double knockout of *NAC SECONDARY WALL THICKENINGS PROMOTING FACTOR 1* (*NST1*) and *NST2 *showed an indehiscent anther phenotype due to failure of secondary wall formation in the anther endothecium (Mitsuda et al., 2005). The double knockout of *NST1* and *NST3/SECONDARY WALL ASSOCIATED NAC DOMAIN PROTEIN 1 *(*SND1*) caused a complete absence of secondary wall in fiber cells of stem and hypocotyl (Mitsuda et al., 2007). The *nst1 nst3* double mutant and the *nst1* single mutant also showed indehiscent siliques caused by the absence of secondary wall in the valve endocarp layer and valve margin (Mitsuda and Ohme-Takagi, 2008). The VNDs and NSTs are related NAC families, forming small adjacent subfamilies in the NAC transcription factor family phylogenetic tree (Mitsuda et al., 2005). Overexpression of these NAC proteins similarly induced ectopic secondary wall formation in various tissues (Kubo et al., 2005; Mitsuda et al., 2005; Zhong et al., 2006). Several MYBs and other transcription factors act downstream of the NACs in a complicated regulatory network (Zhong et al., 2007; Soyano et al., 2008; Zhou et al., 2009; Ko et al., 2009, 2012; Bhargava et al., 2010; Wang et al., 2010; Li et al., 2011; Cassan-Wang et al., 2013). Several orthologous genes in poplar and *Medicago truncatula* (Barrel Medic) also function in secondary wall formation (Zhao et al., 2010; Zhong et al., 2011b). In poplar, a new systematic nomenclature was proposed for the *VND* and *NST* orthologs; according to this nomenclature, the genes would be named *WOOD-ASSOCIATED NAC DOMAIN*s (*WND*s) or *VND, NST/SND AND SMB RELATED PROTEIN*s (*VNS*s) because their expression patterns are not readily classified into either *VND* or *NST* types as in *Arabidopsis* (Ohtani et al., 2011; Zhong et al., 2011b). In monocots, several rice and corn *NST* orthologs, which were renamed *SECONDARY WALL NAC DOMAIN PROTEIN*s (*SWN*s), were recently shown to restore the drooping phenotype of *Arabidopsis nst1 nst3* double mutants and induce ectopic secondary formation when overexpressed in *Arabidopsis *(Zhong et al., 2011a). However, it remains unclear whether *SWN*s actually regulate secondary wall formation in monocots.

In this study, we demonstrate that rice OsSWN proteins can regulate secondary wall formation in rice, and that the expression of a chimeric repressor of *OsSWN2* improves digestibility as forage by causing a reduction of lignin content.

## MATERIALS AND METHODS

### PREPARATION OF PHYLOGENETIC TREE

NAC transcription factor genes similar to *NST1* were identified by TBLASTN searches of all coding sequences (CDSs) of *Arabidopsis* and rice, using NST1 as query. Full-length amino-acid sequence alignment was prepared using MAFFT (Katoh et al., 2002) and the phylogenetic tree was built by the Neighbor-Joining method with MEGA5 (Tamura et al., 2011). The percentage of replicate trees in which the associated taxa clustered together in the bootstrap test (1000 replicates) is shown next to the branches.

### PLASMID CONSTRUCTION

*ProOsACT1:OsSWN1/2S-SRDX,* *35S:OsSWN1/2S* and *ProNST3:*OsSWN1/2S were prepared based on pActSRDXG, p35SG and pNST3_EntG vectors, which were previously described (Mitsuda et al., 2005, 2006, 2007). The CDSs of *OsSWN1* and *OsSWN2S* were amplified with the following primer sets. For *OsSWN1*, OsNST2N (5′-GATGAGCATATCGGTGAACGGGCAGTCGGT-3′), OsNST2C (5′-TACGTTATTCATGGTCGTCAAGTCTGCGTG-3′) and OsNST2S (5′-TCAACGGTCACCGTCGAGCAGTTGCTCGGG-3′). For *OsSWN2S*, OsNST1N (5′-GATGAGCATCTCGGTGAACGGGCAGTCGTG-3′), OsNST1C (5′-TGGCCCAGTCCGTGCGGTGGTGGT-3′) and OsNST1S (5′-TCATGGCCCAGTCCGTGCGGTGGTGGT-3′). Each CDS was ligated into the SmaI sites of the pActSRDXG, p35SG and pNST3_EntG vectors. *ProZmUBQ1:OsSWN1/2S-SRDX* was prepared similarly, based on the pUBQ1SXG vector, which was modified from the pActSRDXG vector. For the cloning of *OsSWN *promoters, the following primer sets were used. For *OsSWN1*, OsNST2pFA3 (5′-AAATTTGGCGCGCTTGCCGACGCTCGGAGCGTGCGAGCTG-3′) and OsNST2proR2Bam (5′-AAATTTGGATCCACCGACTGCCCGTTCACCGATATGC-3′). For *OsSWN2*, OsNST1pFA (5′-AAATTTGGCGCGCCACATATGTTGGATGTATTCTCCGAAA-3′) and OsNST1pRB (5′-AAATTTGGATCCATTGATGATCTTCTTCTTCTTCTCCTT-3′). The amplified fragments were cloned into the AscI/BamHI site of the pSRDX_NOSG vector, which does not have a plant promoter, and into the pGUS_Ent vector for promoter-GUS analysis. The *OsSWN* CDSs were cloned into the SmaI site of promoter-inserted pSRDX_NOSG vectors. The contents of these vectors were transferred into the binary vector pBCKH (Mitsuda et al., 2006) to prepare transgenic *Arabidopsis* and rice. For the reporter plasmid for the transient effector-reporter analysis, two pairs of synthetic oligonucleotides; AscNSTx3Not_F (5′-CGCGCCGTATACCTTGTGAATGAAGAAACTGTATACCTTGTGAATGAAGAAACTGTATACCTTGTGAATGAAGAAACTGC-3′) and AscNSTx3Not_R (5′-GGCCGCAGTTTCTTCATTCACAAGGTATACAGTTTCTTCATTCACAAGGTATACAGTTTCTTCATTCACAAGGTATACGG-3′), AscmSNBEx3Not_F (5′-CGCGCCGTATACAAGGTGAATGAAGAAACTGTATACAAGGTGAATGAAGAAACTGTATACAAGGTGAATGAAGAAACTGC-3′) and AscmSNB-Ex3Not_R (5′-GGCCGCAGTTTCTTCATTCACCTTGTATACAGTTTCTTCATTCACCTTGTATACAGTTTCTTCATTCACCTTGTATACGG-3′) containing three tandem repeats of the binding sequence for NST transcription factors (SNBE; Zhong et al., 2010) or mutated SNBEs were annealed and ligated into the pTATA_LUC_HSP vector, which harbors a TATA box, translational enhancer sequence, firefly luciferase gene and heat shock protein (HSP; Nagaya et al., 2010) terminator sequence. For the OsSWN2L effector plasmid, the CDS of *OsSWN2L* (GenBank accession number JN634071) was artificially synthesized and cloned into the SmaI site of the p35SG vector.

### PLANT CULTIVATION AND TRANSFORMATION

*Arabidopsis thaliana* ecotype Columbia-0 was grown on soil at 23°C, under a 16 h day/8 h night cycle. For the genetic transformation, the floral dip method was employed (Clough and Bent, 1998). *Agrobacterium*-mediated transformation of rice cultivar “Nipponbare” was performed as described previously (Mitsuda et al., 2006). Regenerated rice was grown in soil for rice seedlings under natural sunlight in a closed greenhouse at 22–32°C, transferred to square-shaped plastic pots after 1–2 weeks and cultivated under mostly submerged water conditions (water height from the base of pot: 60–90 mm).

### LIGHT AND FLUORESCENCE MICROSCOPY

For GUS staining, plant tissues were incubated in 100 mM sodium phosphate buffer, pH 7.0, containing 0.1% Triton X-100, 1 mM 5-bromo-4-chloro-3-indolyl-β-D-glucuronide, and 0.5 mM potassium ferricyanide at 37°C for up to 16 h. Stained or harvested leaf tissue was embedded in 5% agar and 100-μm sections were prepared with a vibrating microtome (HM-650V; Microm Inc.). All observations by light and fluorescence microscopy were made with the Axioskop2 plus system (Zeiss Inc.). For observations of lignin autofluorescence, we used a filter with the following specifications: excitation filter: 365 nm short pass; dichroic mirror: 395 nm; emission filter: 400 nm long pass. To observe ectopic secondary wall thickening, we cleared tissues by incubating them overnight in 70% lactic acid at 50°C. To stain lignin specifically, sections were stained with 2% (w/v) phloroglucinol in 95% ethanol for 2–5 min, washed in 10 N HCl for 1 min, and mounted in 5 N HCl.

### TRANSIENT EFFECTOR-REPORTER ANALYSIS

Transient effector-reporter analysis using protoplasts isolated from long-day-grown *Arabidopsis *rosette leaves was performed as described (Fujikawa and Kato, 2007). Briefly, 0.5–1.0 mm strips of sliced leaves were incubated in an enzyme solution containing 1% (w/v) cellulase “onozuka” R10 (Yakult Pharmaceutical Inc., Japan), 0.25% (w/v) macerozyme “onozuka” R10 (Yakult), 0.4 M mannitol, 20 mM MES, 20 mM KCl, 10 mM CaCl_2_, and 5 mM 2-mercaptoethanol for 3 h with shaking at 50 rpm. The solution was then filtered through 75-μm nylon mesh (BD Biosciences Inc., USA) and collected by centrifugation at 100 × *g* for 5 min. The pellet was washed twice with W5 buffer containing 150 mM NaCl, 125 mM CaCl_2_, 5 mM KCl, and 2 mM MES, followed by centrifugation at 100 × *g* for 5 min. The protoplast suspension was kept at 4°C for 30 min. After removing the supernatant, the protoplast cells were resuspended in MMg solution containing 0.4 M mannitol, 15 mM MgCl_2_, and 4 mM MES and then adjusted to 1.5–2.0 × 10^5^ cells/ml. The protoplast suspension, plasmids and transduction solution containing 40% polyethylene glycol, 0.2 M mannitol, and 0.1 M CaCl_2_ were thoroughly mixed with a vortex mixer at 900 rpm for 15 s and then kept at room temperature for 10 min. After introducing the plasmids, the protoplasts were washed three times with W5 buffer and then incubated at 22°C for 18 h in dark. Firefly luciferase driven by a minimal promoter with three tandem repeats of the NST binding sequence or its mutated version was employed as a reporter. The details of its construction are described in the Section “Plasmid Construction.” Along with the reporter construct, 35S:OsSWN1, 35S:OsSWN2S or 35S:OsSWN2L was co-introduced as the effector. As the internal reference, a modified *Renilla* luciferase (*hRLUC*; Promega Inc.) gene driven by the 35S promoter (phRLHSP; Nagaya et al., 2010) was used to normalize the data.

### MEASUREMENT OF YOUNG’S MODULUS

30 mm-long explants were excised from the base of inflorescence stems of 14 2-month-old *Arabidopsis* plants. They were immediately fixed by immersion in 85°C methanol for 5 min and stored in methanol at room temperature. They were rehydrated and treated with 0.2 mg/ml pronase from *Actinomyces *spp*. *(Actinase E, Kaken Pharmaceutical Inc., Japan) in 50 mM potassium-phosphate buffer (pH 7.0) containing 5% ethanol for 18 h after pre-incubation in protease solution for 2 h at 37°C. The protease-treated explants were washed with distilled water and stored in methanol. Force-extension relationships were measured with a tensile-testing instrument (Auto com-200N A/C, TS Engineering Inc., Japan). After measurement, the explant was dried and weighed for calculation of its mass per length. Young’s modulus was calculated by employing Cleland’s formula (Cleland, 1967).

### ANALYSIS OF WATER TRANSPORT

To analyze water transport in *ProOsSWN2:OsSWN2S-SRDX* rice, we employed Fantasy dye solution for the staining of cut leaves (red type, Palace chemical Inc., Japan; Murakami et al., 2006). The petioles of fully developed rice leaves were immersed in the Fantasy dye solution and observed for up to 120 minutes. After staining, leaves (0.07–0.35 g fresh weight) were cut into ca. 3 cm-length pieces, fixed and stored in 5 ml FAA solution [formaldehyde, acetic acid, and ethanol (8:1:1 v/v)] at room temperature. Most of the red dye was extracted from the leaf tissue with FAA in a week. The absorption spectra of the extracts were scanned and we found the absorbance at 550 nm was specific for extracts from cut leaves. The absorbance at 625 nm was used as a blank in each extract. Delta absorbance (550–625 nm) was converted into arbitrary and quantitative units of dye uptake by leaves with a standard curve, *y* (units/ ml) = 0.578 × delta absorbance - 0.0072.

### TEM IMAGING AND ANALYSIS

Short pieces of rice leaf blades were fixed in 30 mM HEPES containing 2% paraformaldehyde and 2% glutaraldehyde then fixed in HEPES containing 2% osmium tetroxide. Fixed tissues were embedded in Q651 resin (Nissin EM Inc., Japan). Sections of 80–90 nm thick were post-stained with uranyl acetate and lead citrate and observed with a JEM1200EX transmission electron microscope (JEOL Inc., Japan) at an accelerating voltage of 80 kV. Thickness of secondary walls in several layers of cortical fiber cells beneath the epidermis was measured for more than 10 cells in each of 7–9 pictures.

### THE ANALYSIS OF SACCHARIFICATION RATE, LIGNIN CONTENT AND ACID DETERGENT FIBER

Culms of rice were collected after the harvest of mature seeds and dried at 70°C for 1 week. The amylase-treated sample was prepared as follows. The samples were ground and the resulting powder (100 mg) was treated with amylase solution (3 U/ml of α-Amylase from porcine pancreas and 30 U/ml of amyloglucosidase from *Aspergillus niger *in 0.1 M sodium maleate buffer at pH 6.0) at 37°C for 16 h with shaking. After amylase treatment, the sample was washed with ethanol and water, then dried at 65–70°C for 3 days and designated as the amylase-treated sample. The saccharification rate of the amylase-treated sample was measured by enzymatic reaction with Celluclast 1.5 l and Novozyme 188 (Novozymes Inc., Japan) as previously described (Iwase et al., 2009).

We quantified lignin content by the acetyl bromide method, following the protocol of Johnson et al. (1961) modified by Morrison (1972); Iiyama and Wallis (1988) and Hatfield et al. (1999). The weight of the amylase-treated sample (1.00–2.00 mg per tube) was measured in glass culture tubes (16 × 100 mm) fitted with Teflon-lined screw caps. 0.5 ml of freshly prepared acetyl bromide reagent [25% (v/v) acetyl bromide (Wako Inc., Japan) in glacial acetic acid] was added and heated at 50°C for 2 h. After heating, acetic acid (1.5 ml) was added and the sample was centrifuged at 3000 *g* for 15 min with a swing rotor at room temperature. The supernatant (0.4 ml) was transferred to a 1.5 ml tube that contained 0.3 mL of 0.3 M NaOH and 0.5 mL of acetic acid. After mixing, 0.1 ml of 0.5 M hydroxylamine was added to each tube and the absorbance at 280 nm was measured. Lignin treated with alkali carboxylate (Sigma Inc., USA) was used as a standard for lignin content. All measurements were run in triplicate for each sample.****

Digestibility as forage was analyzed by a modified method as described previously (Abe, 1988). Approximately 0.5 g of sample was boiled with 10 ml of distilled water in a screw cap bottle for 1 min on a hot plate to denature starch. After cooling, 35 ml of enzyme solution consisting of 0.2% cellulase and 0.01% α-amylase in 0.1 M acetate buffer was added and incubated at 40°C with shaking for 17.5 h. The sample was filtered through filter paper (Toyo 5A, 125 mm) and the residue was washed three times with distilled water. The residue in the filter paper was dried at 105°C overnight and weighed after cooling in a desiccator. Digestibility of the sample was calculated by the following equation:****100 - 100(A′ - A)/S(G/100). S, A, G, A′ represent weight of the sample, weight of container + filter paper, dry matter percentage and weight of container + filter paper + residue, respectively.****

Acid detergent fiber (ADF) was analyzed according to Van Soest (1963). 50 ml of acid detergent (AD) solution consisting of 1 N H_2_SO_4_ and 2% (w/v) CTAB was added to about 0.5 g of sample and placed on the heating unit for 60 min, after which the solution was filtered through a fritted glass. After washing with distilled water and acetone, the fritted glass crucible with residue was dried at 105°C overnight. After cooling in a desiccator and weighing, the crucible with residue was placed at 500°C for 3–4 h in a muffle furnace. After cooling, the ash was weighed. ADF was calculated by the following equation: 100(A - B)/S(G/100). S, A, B and G represent weight of the sample, weight of the crucible + residue, weight of the crucible + ash and dry matter percentage, respectively.****

### SUGAR COMPOSITION ANALYSIS

Matrix polysaccharides of cell wall in culm sample (3–5 mg) treated with α-amylase and amyloglucosidase were hydrolyzed in 1 ml of 2 M trifluoroacetic acid (TFA) at 121°C for 60 min (Albersheim et al., 1967). After cooling to room temperature, the hydrolysate (TFA soluble fraction) was separated from TFA insoluble residues by centrifugation at 2000 × *g* for 5 min using a swing rotor and then dried at 80°C with N_2_ aeration. The dried sugars were dissolved in distilled water for the analysis of sugar composition. The TFA insoluble residues were rinsed with distilled water and ethanol and then completely dried at 80°C for 24 h. The dried residue was treated with 200 μl of 72% (w/w) sulfuric acid and incubated for 1 h at room temperature with agitation. Then the mixture was diluted into 4 ml of distilled water and autoclaved at 121°C for 1 h. Sulfuric acid hydrolysate (TFA insoluble fraction) was neutralized with calcium carbonate and then used for the sugar composition analysis. Mono-saccharide composition of each hydrolysate was analyzed by Shimadzu Prominence HPLC system (Shimadzu Inc., Japan) with fluorescence detector (RF-AXL, Shimadzu) as described by Kawai et al. (2012). The standard sugars used in this study were glucose, xylose, arabinose, rhamnose, galactose, and fucose (Wako Pure Chemical Industry Inc., Japan). Samples were separated with Asahipak NH2P-50 4E (Showa Denko Inc., Japan) with guard column Asahipak NH2P-50G 4A (Showa Denko Inc.) and Opti-guard DVB (Lab Lab Company Inc., Japan) under 45°C at a flow rate of 0.8 ml/min. The mobile phase was 0.3% phosphoric acid:acetonitrile (15:85 v/v).

## RESULTS

### *OsSWN* PROMOTERS ARE ACTIVE IN RICE XYLEM CELLS

**FIGURE 1:**
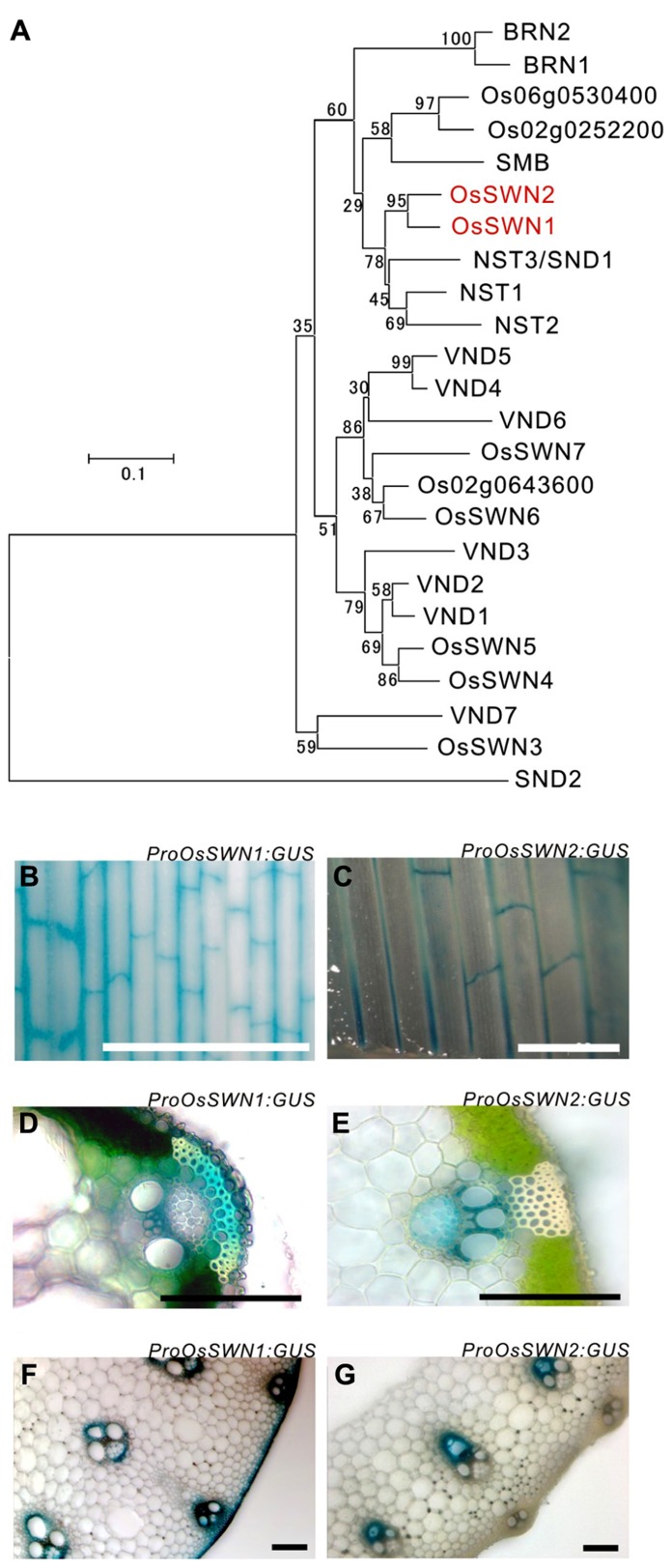
***OsSWN1* and *OsSWN2 *promoters are active in the tissues where secondary walls develop.** **(A)** Neighbor-joining phylogenetic tree of OsSWN proteins and their homologous proteins in rice and *Arabidopsis*. The number on each branch represents percentage of bootstrap values among 1000 tests. **(B**–**G)** GUS stained tissues of *ProOsSWN1:GUS* rice **(B, D, F)** and *ProOsSWN2:GUS* rice **(C, E, G)**. Leaf **(B, C)**, section of leaf **(D, E)** and section of culm **(F, G)**. Bars represent 1 mm in **(B)** and **(C)**, 100 μm in **(D–G)**.

To analyze how secondary wall formation in rice is regulated and to determine which promoter is most suitable for the manipulation of rice secondary walls, we surveyed rice NAC transcription factor genes that are similar to *Arabidopsis* *NST*s, which act as master regulators of secondary wall formation. We found two close orthologous genes (**Figure 1A**), *OsSWN1* (*Os06g0131700*) and* OsSWN2 *(*Os08g0115800*), which were previously reported by Zhong et al. (2011a). We tested the promoter activities of these genes using β-glucuronidase (*GUS*) as a reporter, generating transgenic rice expressing *ProOsSWN1:GUS *and *ProOsSWN2:GUS*. The approximately 3 kbp promoter regions of both *OsSWN1 *and *OsSWN2 *were active in leaf veins but examination of leaf cross sections revealed slight differences in their expression patterns (**Figures 1B–G**). The *OsSWN2* promoter was particularly active in bundle sheath, including vascular vessels in the xylem (**Figures 1C,E,G**) and the *OsSWN1* promoter was preferentially active in several layers of sclerenchymatous cells beneath the epidermis in addition to the bundle sheath (**Figures 1B,D,F**). This pattern of *OsSWN1* promoter activity was consistent with the previously reported *in situ* RNA hybridization data (Zhong et al., 2011a).

### OsSWN1 AND OsSWN2L, BUT NOT OsSWN2S, CAN ACTIVATE SECONDARY WALL FORMATION IN *ARABIDOPSIS*

**FIGURE 2:**
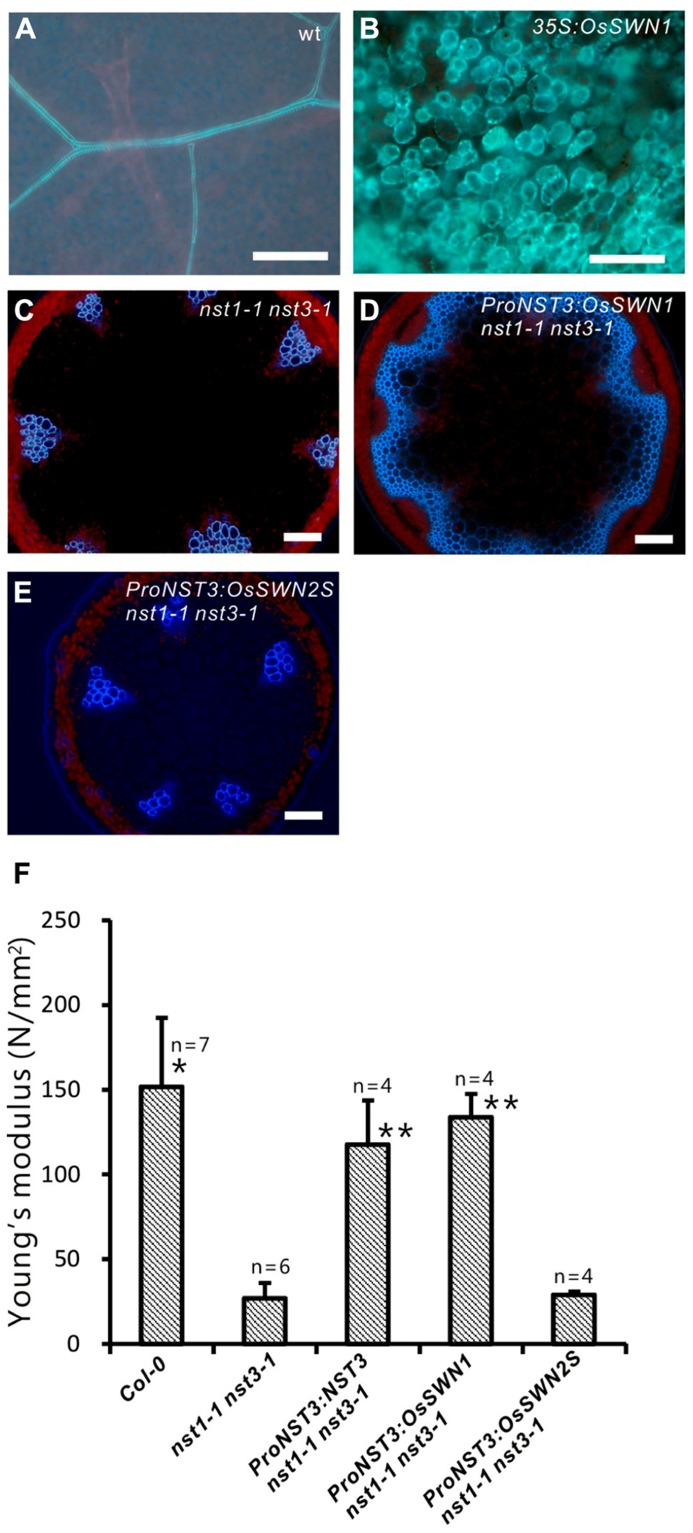
**OsSWN1 but not OsSWN2S restored the phenotype of *nst1-1 nst3-1*.** **(A–E)** Fluorescence images under UV illumination. Blue auto-fluorescence represents lignin. Wild-type leaf **(A)**, leaf of *35S:*OsSWN1 Arabidopsis **(B)**, cross section of stem of *nst1-1 nst3-1* double mutant **(C)**,* nst1-1 nst3-1 ProNST3:OsSWN1 ***(D)** and* nst1-1 nst3-1 Pro*NST3:OsSWN2S Arabidopsis **(E)**. Bars represent 100 μm. **(F)** Comparison of physical strength (Young’s modulus) of inflorescence stem. “*” and “**” indicate values significantly different from the *nst1-1 nst3-1 *mutant by Welch’s *t*-test with *P* < 0.05 and 0.01, respectively.

Because the *OsSWN1* and *2* promoters were active in tissues where secondary walls develop, we next tested whether the OsSWN1 and 2 proteins could activate secondary wall formation. As previously shown, overexpression of *NST1*, *NST2* and *NST3* induced ectopic deposition of secondary wall in various aboveground organs of *Arabidopsis* (Mitsuda et al., 2005, 2007). To test the ability of OsSWN proteins to induce secondary wall deposition, we obtained cDNAs for each protein (AK119784 for *OsSWN1*; AK109860 and JN634071 for *OsSWN2*; The Rice Full-Length cDNA Consortium, 2003; Satoh et al., 2007). We found that *OsSWN2* produces two alternatively spliced forms that encode proteins of different lengths. The predicted protein from the AK109860 cDNA (*OsSWN2S*) is shorter than the predicted protein from the JN634071 cDNA (*OsSWN2L*) and seems to lack the transcriptional activation domain. OsSWN2L was not described in the most recent study (Zhong et al., 2011a) but was recently registered in GenBank. We made overexpression constructs for *OsSWN1* and *OsSWN2S*, and transformed *Arabidopsis* with these constructs. Overexpression of *OsSWN2S* in *Arabidopsis* did not induce any ectopic secondary wall formation, but overexpression of *OsSWN1* strongly induced ectopic secondary wall formation in *Arabidopsis* (**Figures 2A,B**; Zhong et al., 2011a). We also transformed *OsSWN* expression constructs into *nst1-1 nst3-1* mutants, using *OsSWN* cDNAs expressed under the control of the* NST3* promoter, which is preferentially active in the fiber cells of the inflorescence stem. We found that, consistent with the results of ectopic expression, OsSWN1, but not OsSWN2S, induced secondary wall formation and restored the fragile-stem phenotype of *nst1-1 nst3-1* double knockout *Arabidopsis* plants when it was expressed under the control of the* NST3* promoter (**Figures 2C–F**).

**FIGURE 3:**
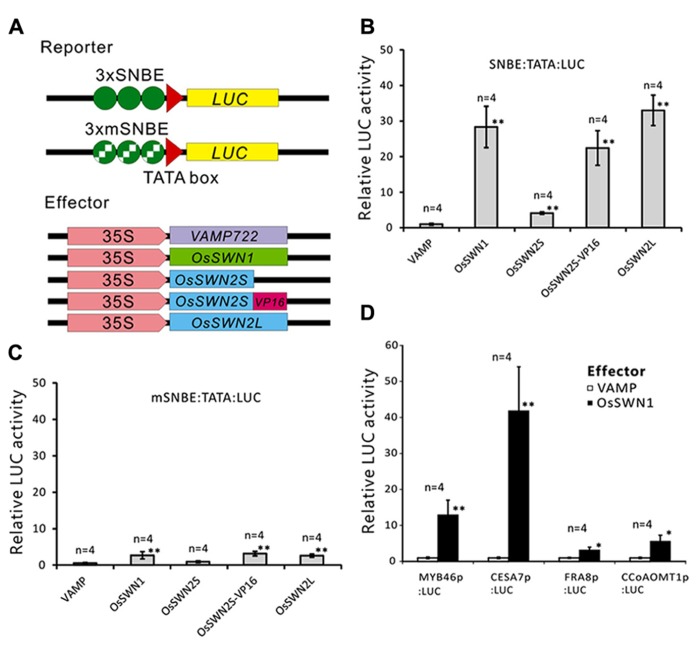
**OsSWN1 and OsSWN2L but not OsSWN2S can activate transcription. (A)** Schematic of constructs used in the reporter-effector transient assay in *Arabidopsis* leaf protoplasts. “SNBE” and “mSNBE” represent the binding sequence for NST transcription factors and a mutated version of the binding site, respectively. “LUC” represents firefly luciferase, which was used as the reporter in this experiment. VAMP722, which localizes to the vacuolar membrane (Uemura et al., 2004), was employed as the negative control. **(B, C)** Relative LUC activity of the reporter constructs shown in **(A)** when each effector was cotransfected into *Arabidopsis* leaf protoplasts. LUC activity when the negative control was cotransfected was set to 1 in **(B–D)**. **(D)** Relative LUC activity of the reporter constructs indicated on the *X* axis when *35S:OsSWN1* or *35S:VAMP722 *was cotransfected into *Arabidopsis* leaf protoplast. Error bars indicate SD. “*” and “**” indicate values significantly different from the negative control by Welch’s *t*-test with *P* < 0.05 and 0.01, respectively.

We next directly tested whether OsSWN1 and 2S/L can activate transcription. Based on its ability to induce ectopic secondary wall formation and to rescue the *nst1-1 nst3-1* mutant, we hypothesized that OsSWN1 may act as a strong activator. Also, based on its length, OsSWN2L may act as an activator, but OsSWN2S likely lacks the ability to activate transcription. To test the activation activities of OsSWN1 and OsSWN2S/L, we performed transient effector-reporter experiments using *Arabidopsis* protoplasts derived from rosette leaves. For the reporter, we used a construct with three repeats of the SNBE sequence, which was previously reported to be a consensus binding site for NST transcription factors (Zhong et al., 2007) and we used a mutated sequence (mSNBE) as a negative control (**Figure 3A**). As expected, the *OsSWN1* effector activated the expression of the reporter gene by as much as 20-fold, and the *OsSWN2L *effector activated the reporter more than 30-fold (**Figure 3B**). By contrast, *OsSWN2S* showed very weak activation compared with the control effector (VAMP722; **Figure 3B**). However, when *OsSWN2S* was fused with the VP16 activation domain derived from herpes simplex virus (*OsSWN2S-VP16*), the effector activated the reporter gene more than 20-fold (**Figure 3B**). In the negative control, OsSWN1, OsSWN2S-VP16 and OsSWN2L showed very little activation of reporter expression driven by the repeated mSNBE (**Figure 3C**). These results indicate that OsSWN1, OsSWN2S and OsSWN2L bind to the SNBE sequence specifically but OsSWN2S does not have adequate activation activity. We further tested whether OsSWN1 can activate native promoters of *Arabidopsis* genes that are known to function downstream of NST transcription factors. As shown in **Figure 3D**, OsSWN1 activated promoters of *MYB46*, *CELLULOSE SYNTHASE A 7* (*CESA7*), *FRAGILE FIBER 8* (*FRA8*) and *Caffeoyl-CoA 3-*O*-methyltransferase* *1* (*CCoAOMT1*), suggesting that OsSWN1 induces secondary wall formation by a mechanism similar to that of *Arabidopsis* NST transcription factors.

### THE OsSWN2S CHIMERIC REPRESSOR ALTERS SECONDARY WALL FORMATION IN RICE

**FIGURE 4:**
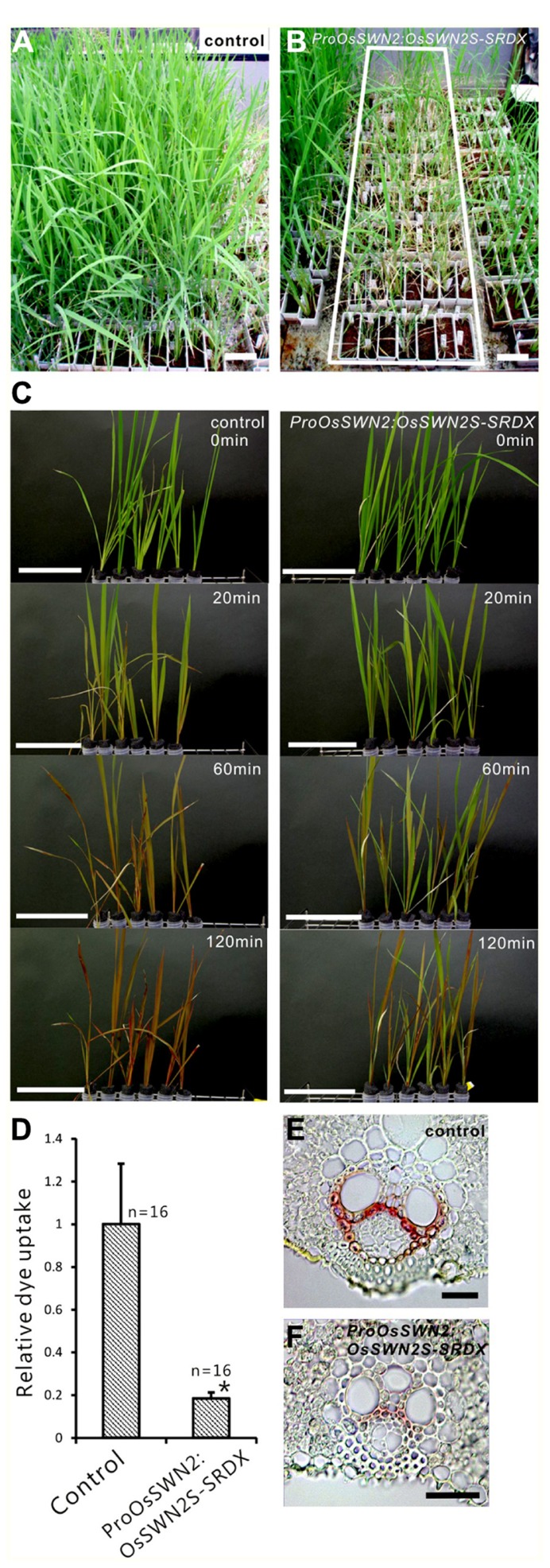
***ProOsSWN2:OsSWN2S-SRDX *rice plants are defective in water transport.** **(A)** *ProOsSWN2:GUS* rice plants (control). **(B)** *ProOsSWN2:OsSWN2S-SRDX* rice plants, indicated by the white rectangle. **(C)** Time-course examination of water uptake of shoots excised from the *ProOsSWN2:OsSWN2S-SRDX* plants before the onset of leaf rolling (1–2 weeks culture after budding). Water with a red tracer dye was used in the experiment. Bars in **(A–C)** represent 10 cm. **(D)** Relative dye uptake by *ProOsSWN2:OsSWN2S-SRDX *plants compared to control plants (set to 1). Error bars represents standard error. “*” indicates values considered to be significantly different from control by Welch’s *t*-test with *P* < 0.05. **(E, F)** Cross sections of leaf of control plants **(E)** and *ProOsSWN1:OsSWN2S-SRDX* rice plants **(F)** stained with phloroglucinol to detect lignin. Bars in **(E, F)** represent 100 μm.

To analyze the role of *OsSWN* genes in rice and examine their potential utility for cell wall engineering in rice, we prepared transgenic rice lines that express chimeric repressors (OsSWNs-SRDX), which consist of each *OsSWN* fused with the modified plant specific repression domain [ethylene-responsive element binding factor-associated amphiphilic repression (EAR) motif] derived from *SUPERMAN* (SRDX: LDLDLELRLGFA; Hiratsu et al., 2003). The resulting chimeric repressor is expected to work as a strong dominant-negative transcription factor that represses the expression of its target genes and induces a loss-of-function phenotype (Hiratsu et al., 2003). Neither *OsSWN1-SRDX* nor *OsSWN2S-SRDX *induced apparent abnormalities when expressed under the control of promoters derived from the rice *actin* gene or the maize *ubiquitin* gene. We cannot exclude the possibility that the transgene was not properly expressed in the plants because we did not examine transgene expression. However, our results are consistent with results in *Arabidopsis*, in which the CaMV 35S promoter, which induces ubiquitous expression, was not efficient in inducing a loss-of-function phenotype in fiber cells with a *NST1-SRDX* construct (Mitsuda et al., 2005, 2007). Therefore we next tested the *OsSWN2* promoter, which is preferentially active in the vascular bundle (**Figures 1B,D,F**), and found that *ProOsSWN2:OsSWN2S-SRDX* caused severe retardation of shoot growth and rolling of vegetative leaves, resulting in needle-like leaf shape, 4–6 weeks after culture under normal water conditions. In addition, the rolled leaves of *ProOsSWN2:OsSWN2S-SRDX* lines turned brown at the maximum tillering stage. We describe this phenotype of *ProOsSWN2:OsSWN2S-SRDX* as “para-wilting”; the para-wilting phenotype was observed in 499 out of 568 *ProOsSWN2:OsSWN2S-SRDX* transgenic plants but never observed in 779 control transgenic plants expressing the *ProOsSWN2:GUS *construct and 396 *ProOsSWN2:OsSWN1-SRDX* transgenic plants (**Table 1**, **Figures 4A,B**).

**Table 1:** Para-wilting phenotype of the *ProOsSWN2:OsSWN2S-SRDX* rice plants.

Transgenic population	Number of plants showing para-wilting^*1^	Total number of hygromycin-resistant plants	Percentage showing para-wilting (%)
***ProOsSWN2:GUS***
1	0	228	0
2	0	207	0
3	0	145	0
4	0	199	0
Total	0	779	0 ± 0^*2^
***ProOsSWN2:OsSWN2S-SRDX***
1	163	177	92.1
2	155	182	85.2
3	181	209	86.6
Total	499	568	88 ± 2.6^*2^
***ProOsSWN2:OsSWN1-SRDX***
1	0	396	0
Total	0	396	0

*1 Para-wilting: leaves rolling into needle-like shape and browning under normal water conditions. Number of plants showing para-wilting was determined after 18–24 weeks of cultivation in the greenhouse. Leaves started to roll after 4–6 weeks of cultivation.

*2 Mean ± SE (*n* = 3 or 4).

Because the *OsSWN2* promoter is active in the vascular bundle, we examined water transport in the *ProOsOSWN2:OsSWN2S-SRDX *plants using a tracer, red Fantasy dye solution. Even though no morphological abnormalities were observed in *ProOsSWN2:OsSWN2S-SRDX* rice leaves at 1–2 weeks after cultivation in the greenhouse, the excised shoots from transgenic plants did not absorb water as fast as the those from control transgenic plants (**Figure 4C**). Quantification of dye uptake supported this observation (**Figure 4D**). These data strongly suggest that water transport was severely impaired in these plants. This might be partly related to incomplete development of vascular vessels, as shown previously in *Arabidopsis* plants that expressed *35S:VND7-SRDX* (Kubo et al., 2005), since detailed examination of cross sections of leaf blades revealed less accumulation of lignin in the transgenic plants (**Figures 4E,F**).

**FIGURE 5:**
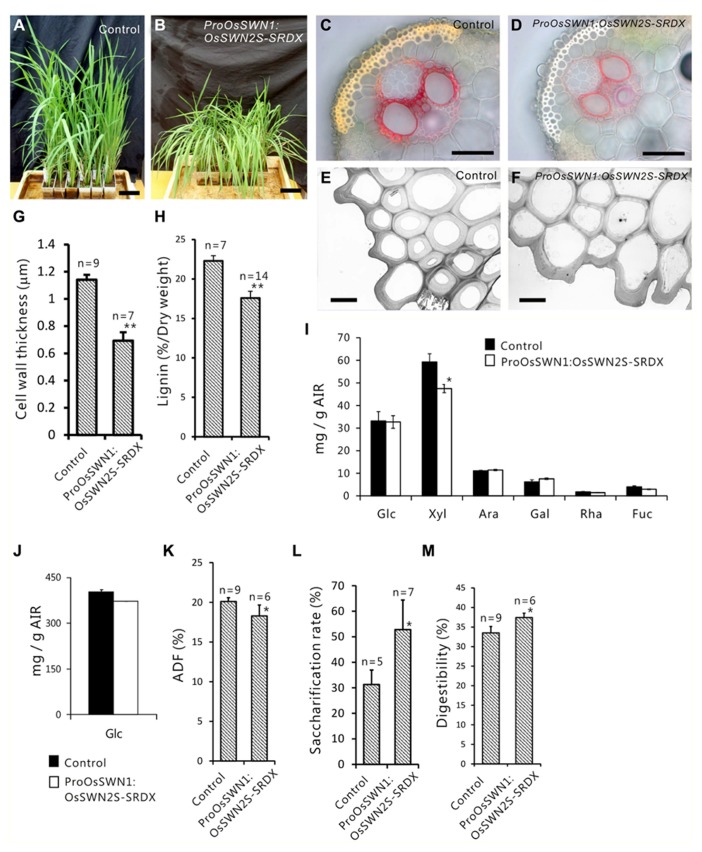
**Reduced secondary cell walls in* ProOsSWN1:OsSWN2S-*SRDX rice.** **(A)** *ProOsSWN1:GUS* rice plants (control). **(B)** *ProOsSWN1:*OsSWN2S-SRDX rice plants. **(C–F)** Cross sections of leaves of control plants **(C, E)** and *ProOsSWN1:OsSWN2S-SRDX* rice plants **(D, F)** stained with phloroglucinol to detect lignin **(C, D)** or observed by transmission electron microscopy **(E, F)**. **(G, H, K–M)** Thickness of secondary walls **(G)**, lignin content **(H)**, the ADF value **(K)**, saccharification rate **(L)** and digestibility as forage **(M)** of *ProOsSWN1:GUS* rice plants (control) and *ProOsSWN1:OsSWN2S-SRDX *rice plants. **(I, J)** Cell wall sugar composition of TFA soluble fraction **(I)** and TFA insoluble fraction **(J)** in alcohol insoluble residues (AIR). Sugar composition was analyzed in AIR of *ProOsSWN1:GU*S [control (black, *n* = 5)] and *ProOsSWN1:OsSWN2S-*SRDX (white, *n* = 11). “*” and “**” indicate *P* value of Welch’s *t*-test <0.05 or 0.01, respectively. Error bars indicate SD in **(G, H, K–M)**, SE in **(I)** and **(J)**. Bars represent 10 cm in **(A)** and **(B)**, 50 μm in **(C)** and **(D)**, 5 μm in **(E)** and **(F)**.

Next, we expressed the chimeric repressors under control of the *OsSWN1* promoter, which is preferentially active in several layers of sclerenchymatous cells beneath the leaf blade epidermis and shows little activity in vascular vessels. Because OsSWN2S-SRDX effectively induced abnormalities in the experiment described above, we focused on OsSWN2-SRDX in this experiment. 192 out of 471 *ProOsSWN1:OsSWN2S-SRDX* rice plants exhibited an apparent drooping phenotype (**Table 2**, **Figures 5A,B**), a slight decrease in height (length of leaves). Detailed examination of leaf cross sections of the *ProOsSWN1:OsSWN2S-SRDX* rice plants revealed a reduction of secondary wall thickenings in the sclerenchymatous cells that form cortical layers beneath the epidermis and in bundle sheath fibers surrounding vascular vessels in xylem (**Figures 5C–F**). Moreover, transmission electron microscopy showed that the secondary walls were thinner in the transgenic rice plants (**Figure 5G**). Staining of the cross sections with phloroglucinol, which specifically stains lignin polymers, revealed reduced accumulation of lignin in the *ProOsSWN1:OsSWN2S-SRDX* rice plants (**Figures 5C,D**). We quantified the lignin content of culms by the acetyl-bromide method and found that it was reduced more than 20% in the transgenic plants compared to the control plants (**Figure 5H**). We further examined the neutral sugar compositions of the culms using liquid chromatography and found that TFA-soluble xylose was significantly reduced, but TFA-soluble arabinose presumably derived from arabinoxylan was not changed (**Figures 5I,J**), suggesting that xylan content was reduced in the cell walls of the *ProOsSWN1:OsSWN2S-SRDX* rice plants. Because some cultivars of rice and related monocot species are consumed as forage, we examined the ADF value, which shows the digestibility of the plant, with higher numbers indicating a higher lignin and crystalline-cellulose content, and therefore lower digestibility. Indeed, the ADF value was also significantly reduced in the *ProOsSWN1:OsSWN2S-SRDX* rice plants (**Figure 5K**). Also, the enzymatic saccharification rate of the cellulosic material was higher in the *ProOsSWN1:OsSWN2S-SRDX* rice plants than in the control transgenic plants (**Figure 5L**). The digestibility as forage was also significantly enhanced in the *ProOsSWN1:OsSWN2S-SRDX* transgenic rice (**Figure 5M**). These data suggest possible mechanisms for future genetic improvement of monocotyledonous forage and bioenergy species using this gene construct.

**Table 2:** Drooping leaf phenotype of the *ProOsSWN1:OsSWN2S-SRDX* rice plants.

Transgenic line	Number of plants that showed drooping leaves	Total number of hygromycin-resistant plants	Percentage with drooping leaf-phenotype (%)
***ProOsSWN1:GUS***
Event no.	0	231	0
1			
2	0	193	0
3	0	255	0
4	0	166	0
Total	0	845	0 ± 0^*^^1^
***ProOsSWN1:OsSWN2S-SRDX***
Event no.	103	204	50.5
1			
2	47	108	43.5
3	42	159	26.4
Total	192	471	40.1 ± 8.8^*^^1^

* Mean ± SE (*n* = 3 or 4)

## DISCUSSION

In this study, to explore the possibility of genetic engineering to improve the cell wall of monocots, we examined rice orthologs of the *Arabidopsis* NST transcription factors that regulate secondary wall formation. We found that *OsSWN1* and *OsSWN2* are expressed in cells where secondary cell walls are formed and can alter secondary cell wall formation in rice. Ectopic expression of OsSWN1 induced secondary wall formation in *Arabidopsis* and OsSWN2S chimeric repressors driven by the *OsSWN1* promoter reduced secondary wall formation in rice. These results might reflect their abilities to activate transcription and we found that OsSWN1 can strongly activate transcription. However, OsSWN2S does not activate transcription, even though it can bind to the same *cis*-element as OsSWN1. It is likely that OsSWN1-SRDX did not function as a repressor in our assays because OsSWN1 itself can strongly activate transcription; this may counteract the negative activity of SRDX. The extent of ectopic secondary wall formation was greatest in the *Arabidopsis* plants overexpressing *OsSWN1*, compared to the other transgenic lines tested. In addition, we used the SRDX sequence, which is mainly used in *Arabidopsis*, and which may not function well in rice. However, OsSWN2S-SRDX efficiently suppressed secondary wall formation, probably because OsSWN2S has no endogenous transcriptional activation activity in our assays to counteract SRDX function. By contrast, OsSWN2L, the recently identified longer version of OsSWN2, has comparable transcriptional activation activity to OsSWN1. These results suggest that either: (i) OsSWN2L functions *in planta *but OsSWN2S does not function, or (ii) OsSWN2L promotes, and OsSWN2S inhibits secondary wall deposition *in planta* to fine-tune the formation of secondary wall, similar to a mechanism previously suggested in poplar (Lin et al., 2012).

OsSWNs clearly do not belong in the VND subgroup, which functions in the formation of vascular vessels, but rather are classified in the NST subgroup, which mainly functions in the fiber cells in *Arabidopsis*. However, the *OsSWN* promoters are active in cells that will form vascular vessels. For example, the rice plants expressing *OsSWN2S-SRDX* driven by the *OsSWN2* promoter showed a severe defect in water transport. There are several rice genes that apparently belong to the VND subgroups, are strongly expressed in vascular bundles, and can restore the drooping phenotype of *nst1 nst3* double mutants (Zhong et al., 2011a). This supports the idea that OsSWN1/2 should not be classified as a VND protein, even if the *OsSWN2* promoter is highly active in vascular vessels. As suggested previously for poplar (Ohtani et al., 2011; Zhong et al., 2011b), the classification of NST and VND subgroups may also be ambiguous in rice.

Reducing the recalcitrance of plant material is emerging as a key issue for plant biotechnology in this decade, both for production of lignocellulosic bioethanol and for digestibility of forage. The *ProOsSWN1:OsSWN2S-SRDX* transgenic rice line developed in this study showed reduced lignin content, enhanced forage digestibility and increased saccharification rate. This result is consistent with the previous finding in *Arabidopsis* that the *nst1-1 nst3-1* double mutant and *ProNST3:NST1-SRDX *transgenic plants showed an increased saccharification rate (Iwase et al., 2009). In *Arabidopsis*, even though the total glucan content was not reduced, the *ProNST3:NST1-SRDX* plants could not stand erect at all and were quite fragile. The transgenic plants generated here show reduced lignin and xylose content but were more sturdy. Rice contains a large amount of silica, which might help support plants with reduced lignocellulose and the transgenic plants also contained residual lignocellulose, as a result of incomplete suppression by OsSWN-SRDX.

The monocot secondary wall is not conspicuous, but it is unequivocally important for bioethanol production and forage quality. In this study, we demonstrated that two OsSWN transcription factors regulate secondary wall formation in rice and therefore may be useful to improve cell wall quality in monocot crops (lower contents of lignin and xylan). We expect further studies of the secondary wall in monocots will improve our understanding of plant cell wall formation, structure, and function and allow key improvements for forage and biofuel crops.

## Conflict of Interest Statement

The authors declare that the research was conducted in the absence of any commercial or financial relationships that could be construed as a potential conflict of interest.
